# Internal Orifice Alloy Closure—A New Procedure to Treat Anal Fistula

**DOI:** 10.3389/fsurg.2022.881060

**Published:** 2022-05-17

**Authors:** Ming Li, Xiaoli Fang, Jun Zhang, Heng Deng

**Affiliations:** ^1^ Department of Anorectal surgery, First Affiliated Hospital of Anhui University of Traditional Chinese Medicine, Hefei, China; ^2^ Department of Anorectal surgery, Second Affiliated Hospital of Anhui University of Traditional Chinese Medicine, Hefei, China

**Keywords:** anal fistula, sphincter-saving technique, internal orifice alloy closure, ultrasound, internal orifice

## Abstract

**Background:**

The internal orifice plays an important role in the pathogenesis and treatment of the most complex fistula-in-ano. The treatment of the internal orifice is considered to be the key to the success of anal fistula surgery. The objective of this study is to evaluate the feasibility of a new sphincter-sparing surgical approach for anal fistula.

**Materials and Methods:**

All hospitalized anal fistula patients were included in this study. Preoperative anorectal ultrasound was done on all the patients. Transanal internal orifice alloy closure (IOAC) was performed through a disposable titanium nickel alloy anal fistula stapler. The external sphincter was not cut. An anal fistula brush was used to curette and clean fistulas. Postoperative anorectal color ultrasound was used for evaluation 2 months postoperatively.

**Results:**

Twenty-one patients (male/female: 18/3, age: 39.7 ± 10.5 years) with fistula-in-ano were included (follow-up: 6–11 months).In total, 38.1% (8) had multiple tracts, and 9.5% (2) belonged to a high anal fistula. In total, 23.8% (5) of anal fistula patients were complicated by Crohn’s disease. The fistula healed completely in 85.7% (18/21) and did not heal in 14.3% (3/21). Three patients who did not heal had conventional surgery reperformed and eventually healed. Except for three patients undergoing additional traditional anal fistula surgery, the Wexner incontinence scores of other patients did not change after surgery compared with before surgery.

**Conclusions:**

IOAC is a novel sphincter-saving technique that is simply effective in treating anal fistula containing Crohn’s anal fistula.

## Introduction

Anal fistula has always been a problem for surgeons (1). Surgeons are forced to choose between two bad options: an effective treatment of the fistula tract or risk of fecal incontinence (2). To seek therapeutic effects and to maximize the protection of anal function have become the constant pursuit of anorectal doctors.

The internal orifice has been found to play an important role in the genesis, development, and treatment of anal fistula diseases (3). Both anal fistula caused by anal gland infection and anal fistula accompanied by inflammatory bowel disease are believed to originate from anal sinus or ulceration of the intestinal wall (4). Based on this concept, blocking the internal orifice to prevent intestinal bacteria from entering tissues has become a strategy for surgeons to create new procedures (5). However, injecting Salvecoll-E (6), fistula-tract laser closure (7), slide flap (8), and stem cells (9) in the treatment of fistula pay too much attention to the internal orifice and fistulas that had formed but do not pay attention to the operability of the technique. They are difficult for beginners to master. Therefore, the focus seems to be shifting to operational and effective technologies that can better serve more patients.

**Figure 1:**
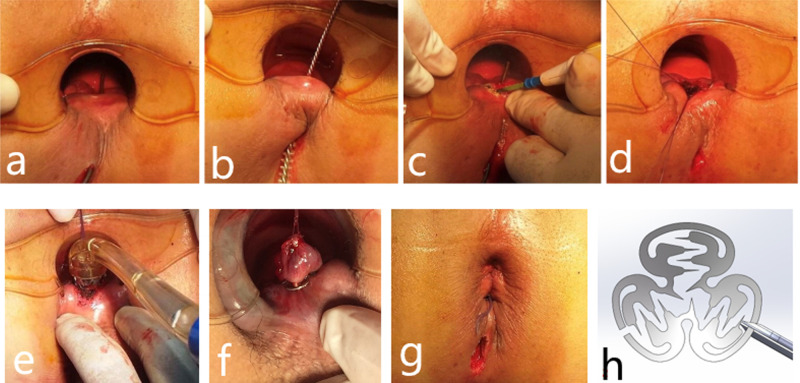
A 36-year-old man with fistula treated by the IOAC procedure with complete healing demonstrated on follow-up anorectal color ultrasound. (**A**–**G**) Procedure for a patient undergoing IOAC surgery and (**H**) alloy anal fistula clamp.

In this study, the use of disposable titanium nickel alloy anal fistula stapler can be easily mastered according to the instructions. An alloy anal fistula clamp has ultrahigh elasticity and continuous centripetal pressure closure of the internal orifice. The internal orifice was kept closed to eradicate the entrance of infection and completely heal the fistula (Figure 1). Because the external sphincter is preserved during surgery, the risk of fecal incontinence is expected to be minimal. The aim of our study was to report treatment results of this simple novel procedure and to explore the factors promoting anal fistula healing.

## Methods

**Figure 2:**
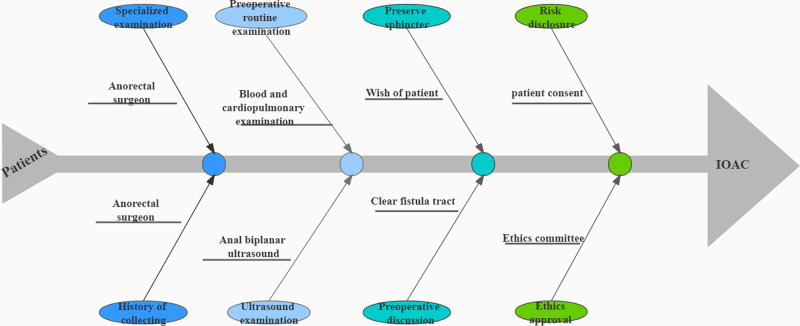
A flow-chart from a patient visit to the surgery. The general course of a patient from a visit to the surgery contains preoperative examination, risk notification, and ethical approval.

A prospective study was performed in which patients operated in our hospital between 1 May 2020 and 1 November 2020 were included. The study was approved by the hospital ethical committee. Each patient was informed of the principle, associated risks, and significance of the study and signed written consent (Figure 2).

### Inclusion Criteria

- The patient was diagnosed with an anal fistula and confirmed by the transcutaneous superficial and transrectal biplanar ultrasound probe.
- The age ranged from 18 to 65 years.

### Exclusion Criteria

- Anal fistula with acute infection.
- Anal fistula with tumor.
- Anal fistula that has been surgically treated.

### Principle behind the Procedure

The fistula-in-ano is the same as other sinus/fistula as it has internal orifices, external orifices, and fistulas. The entry of infection-causing bacteria through the internal orifices is a special feature of anal fistulas. For the fistula to heal completely, the internal orifice needs to be closed up. Traditional resection of the internal orifice, fistula, and external orifice brings a bigger wound, which may cut the external sphincter. On the contrary, internal orifice closure brings a smaller wound and would not damage the external sphincter at all.

Therefore, in the internal orifice alloy closure (IOAC) procedure, the internal orifice is closed up, and the lumen of the fistula is de-epithelialized. For this, the mucosa surrounding the internal orifice is anastomosed with a ring alloy anal clamp, and the fibrotic fistula is also completely destroyed with the fistula brush. This not only shuts off the source of infection but also gives a new chance to the interior of the pipeline, which is allowed to heal by secondary intention.

The main aim of this surgery is to achieve fistula healing without any damage to the external sphincter. This is achieved by removing fibrotic walls and preventing new infections so that the fistula heals well. Postoperatively, the upper end of the pipe is a blind end, there is no fibrous tissue inside the pipe, and the lower end of the pipe is the outer opening for drainage. Inadequate closure of the internal orifice will allow the source of infection to enter from the intestinal tract, leading to nonhealing. Therefore, both the steps are crucial for the success of the operation.

The internal opening is closed by an alloy anal fistula clamp parallel to the mucous membrane to ensure that all mucous membranes are anastomosed. It is also important to move the anal brush up and down repeatedly to ensure the de-epithelialization of the fistula wall.

### Procedure

**Figure 3:**
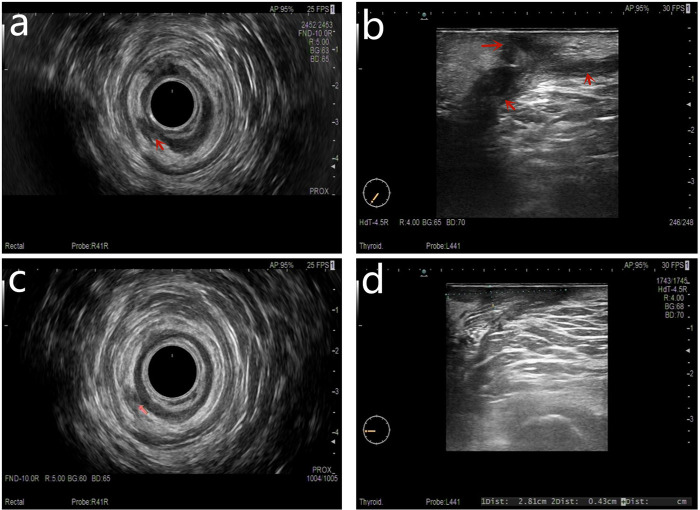
Both preoperative anal fistula and postoperative complete healing were demonstrated on a follow-up anorectal ultrasound scan. (**A**) Preoperation transrectal biplanar probe scans, (**B**) preoperation percutaneous superficial probe scans, (**C**) postoperation follow-up transrectal biplanar probe scans of healed fistula, and (**D**) postoperation follow-up percutaneous superficial probe scans of healed fistula.

All patients underwent anorectal ultrasound examination before surgery. Based on the anorectal ultrasound, the fistula tract was drawn in detail (Figure 3) after a discussion between the ultrasound doctor and the surgeon.

The patient was given combined lumbar epidural anesthesia and then placed in the lithotomy position. An intravenous drip of antibiotics, Ciprofloxacin 500 mg and Ornidazole 500 mg, was given 3 h before the surgery. (1) The internal opening was confirmed by inserting a curved metal probe through the external opening and noticing its egress inside the anal canal. (2) An anal brush was used to clean the epithelial tissue of the inner wall of the fistula. (3) The mucosa of 1 cm around the internal opening was removed. (4) The muscle layer was exposed and cross-sutured into a falling umbrella. (5) The suture tissue was pulled into the head of the release device. (6) Internal opening was anastomosed by releasing the anal fistula clip after exciting the release device. (7) The external opening is enlarged for drainage (Figures 1 **and** 4).

**Figure 4:**
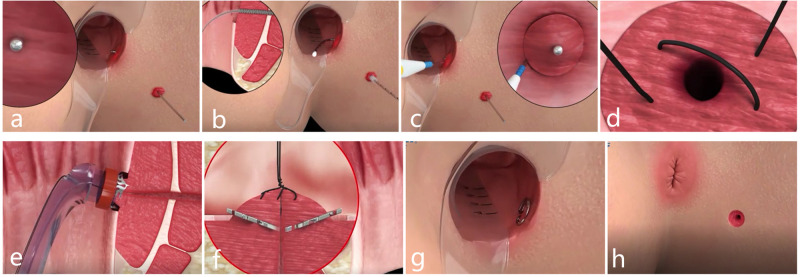
Schematic diagram of the procedure for IOAC. (**A**) Curved metal probe was used to confirm the internal opening. (**B**) Epithelial tissue of the inner wall of the fistula was cleaned by an anal brush. (**C**) Mucosa of 1 cm around the internal opening was removed. (**D**) Muscle layer was exposed and crossed sutured into a falling umbrella. (**E**,**G**) Internal opening was anastomosed by the anal fistula clip. (**H**) External opening was enlarged for drainage.

The clips fall off automatically 4–6 weeks after surgery. If the clips do not fall off, they can be removed. Anorectal ultrasound was again used to assess anal fistula healing (Figure 2). The scrapings and pus was sent for histopathology.

The incontinence was assessed by Wexner incontinence scoring (10) before the surgery and 2 months after the surgery. The six parameters evaluated in the scoring were incontinence to gas, liquid, and solid; the need for padding; antidiarrhea medications; and the ability to defer defecation for 15 min. Zero denotes no incontinence, and 24 denotes total incontinence (11).

The skin of all fistula tracts areas free from tenderness and scarred external orifices, without discharge of pus from any canal or anus, was considered the standard for complete clinical healing of the anal fistula.

### Statistical Analysis

Chi-squared analysis was performed to compare categorical variables. The *t*-test was used to assess continuous variables. *p*  < 0.05 was considered statistically significant.

## Results

Twenty-one patients with fistula-in-ano underwent surgery. The median follow-up was 6 months (range: 5–11 months). The mean age was 39.7 ± 10.5 years, and the male-to-female ratio was 18/3. In total, 33.3% (7) patients had trans-sphincteric anal fistula, and the others had intersphincteric anal fistula. In total, 38.1% (8) had multiple tracts, and 9.5% (2) had high anal fistula. In total, 23.8% (5) of anal fistula patients were complicated by Crohn’s disease. The anal canal wound and the fistula healed completely in 85.7% (18/21) patients; the healing time was 56 ± 8 days after surgery. The anal canal wound and the fistula did not heal in 14.3% (3/21) patients (Table 1). Three patients who did not heal had conventional surgery reperformed and eventually healed at 94, 103, and 108 days after primary surgery, respectively. Anorectal ultrasound also found that the healed fistula emitted a stronger echo than the surrounding tissue when the surgeon considered the anal fistula to be clinically healed.

**Table 1:** Classification of fistula by type, etiology, and relative results.

Number of cases	Type	Beyond anorectum ring	Etiology	Parks typing (intersphincteric/transsphincteric)	Special treatment for etiology	Number of cases of complete healing
*N* = 12	Simple	1	Glandular	11/1	None	12
*N* = 4	Complex	1	Glandular	2/2	None	1
*N* = 1	Simple	0	Crohn’s	1/0	Treatments for Crohn’s disease	1
*N* = 4	Complex	0	Crohn’s	0/4	Treatments for Crohn’s disease	4

The result of the samples sent for histological analysis was chronic inflammatory changes, with a large number of lymphocytes and plasma cells infiltrating, and no tumor cells were found.

The five patients with coexisting Crohn’s disease were treated with different treatments for Crohn’s disease according to their condition. All of them went into remission from Crohn’s disease.

Before the alloy clamp fell off, anal pain gradually decreased with time. When the alloy clamp came off, the pain score was 0. There was no significant bleeding in the anus postoperatively, including during defecation.

Except for three patients undergoing additional traditional anal fistula surgery, the Wexner incontinence scores of other patients did not change after surgery compared with those before surgery. Preoperative incontinence scores were 0.12 ± 0.2, and postsurgery incontinence scores after 3 months were 0.36 ± 0.4 (*p* = 0.24, unpaired *t*-test). No major complications or adverse reactions occurred during the treatment of all patients. In our retrospective analysis, 103 patients who underwent anal fistulotomy, including rubber band tightening during the same period, had varying degrees of anal incontinence, with an incontinence score of 3.93 ± 1.7 at 3 months after surgery (*p* < 0.001, compared with IOAC).

## Discussion

A new surgical procedure for anal fistula, IOAC, has been found by our current study to be simple, inexpensive, effective, and protective of the sphincter.

Although many surgical methods have been innovated in recent years, treating anal fistula is still a huge challenge. Traditional methods such as direct fistula resection and chronic rubber band resection have a high risk of incontinence in these fistulas (12). The newer methods are relatively safe but have a low success rate and are a complicated surgical procedure for a junior surgeon (13). This results in repeated surgeries for patients with anal fistula, which costs a lot of time and money (14). The principles of these methods include closing internal openings and treating fistulas, as well as treating gaps in fistula running. The corresponding surgical names include rectal mucosal muscle flap propulsion repair and anal fistula laser closure (15); anal fistula resection, anatomy, and placement, the latter including intersphincteric fistula ligation (LIFT) (16); video-assisted anal fistula treatment (17); and transanal incision of the sphincter space [transanal opening of intersphincteric space (TROPIS)] surgery (18). In our retrospective analysis, patients who received IOAC had a significant reduction in incomplete anal incontinence compared with patients who underwent anal fistulotomy in the same period.

Further, IOAC was performed only on the internal and external orifices and tracts, without additional surgical approaches. None of the procedures (rectal mucosal muscle flap propulsion repair and anal fistula laser closure; anal fistula resection, anatomy, and placement; LIFT; and TROPIS) avoids additional damage. Only video-assisted anal fistula treatment avoids additional damage, and pathological tissue is used as the approach (17). Unfortunately, video-assisted anal fistula treatment requires more expensive equipment and more experienced surgeons. The price of the IOAC set is $40, well below the cost of consumables used in other technologies.

The LIFT procedure also stops the infection from ligation of the intersphincteric tract. However, theoretically, IOAC might have a higher success rate than LIFT. First, in LIFT, nothing has been done to internal orifices. Second, fibrotic tracts remain after the LIFT procedure. In addition, the over-the-scope clip (OTSC) system is used for the management of leakage after laparoscopic sleeve gastrectomy and treatment of anal fistula (19, 20). Both OTSC clip and IOAC clamp are made of alloy material, and their therapeutic principle is to clamp the rupture of the digestive tract wall. However, first, OTSC is operated under digestive endoscopy for leakage, and the procedure is relatively complicated. Second, in the treatment of anal fistula, the clamp of OTSC has a symmetrical two-blade structure, while the clamp of IOAC has a three-blade structure. The latter has a continuous centripetal force at the internal orifice in terms of mechanics. This makes the latter clamp self-removable or easier to remove in about 6 weeks if it does not fall off by itself.

Detailed analysis of anorectal ultrasound in all the patients helped significantly to track the fistula tracts. This facilitates the treatment of all fistulas by the anal brush.

Although our study has never been reported before, the principle of treatment is also based on closing the internal orifices, managing fistulas, and expanding drainage of the external orifices. This procedure was quite effective (healing rate of 85.7%) in curing fistula-in-ano, including Crohn’s anal fistula and fistula with multiple tracts. Even in the absence of postoperative primary anal fistula healing, additional conventional surgery does not cause more damage or complicate the disease.

To conclude, the success rate of the IOAC procedure in more than 85% of the fistula is quite impressive. In this study, more than 38% had multiple tracts, and more than 23% patients had Crohn’s disease. This procedure has the advantages of a simple operation and no damage to the external sphincter and normal tissues because the surgical approach is in the internal and external orifices. Although the technique is easy to reproduce, it is associated with little pain and early resumption of normal activities. However, therapeutic effects on acute infectious anal fistula are awaited. Retrospective noncomparative studies have many limitations, such as a small number of patients, selective patients, and short follow-ups.

## Data Availability

The raw data supporting the conclusions of this article will be made available by the authors without undue reservation.
