# Supplementary material for: Internal Orifice Alloy Closure—A New Procedure to Treat Anal Fistula
Source: Front Surg. 2022 May 17;9:881060. doi: 10.3389/fsurg.2022.881060 (PMC9157345; doi:10.3389/fsurg.2022.881060)
Supplement: Supplementary file 2 [file Data_Sheet_1_v1.pdf]

（Translation）

Ethics Committee of the First Affiliated Hospital of Anhui University of Chinese Medicine

Ethics review opinions on biomedical research ethics involving human

Li Ming apply for one-off nickel titanium memory alloy anal fistula in internal stapling in anal fistula mouth closed mouth in project research contents involved in the human body, the first affiliated hospital of anhui medical university ethics committee review, the subject conform to the requirements of the involved people biomedical ethical review, agree to conduct research. Approval number:2018AH-25.

Chairman of committee: Hou Yong

2019.04.01

安徽中医药大学第一附属医院伦理委员会

伦理审查批件

伦理审查批件号	2018AH-25
项目名称	一次性钛镍记忆合金肛瘘内口吻合器临床用于肛瘘内口闭合有效性和安全性的随机、单盲、阳性平行对照临床试验
申办者	陕西福泰医疗科技有限公司
研究单位	安徽中医药大学第一附属医院
主要研究者	李明
会议审查时间及审查委员	2019 年 3 月 6 日　审查委员见会议签到表
审查地点	合肥梅山路 117 号安徽中医药大学第一附属医院
审查类别/审查方式	初始/会议审查　复审/快速审查
批准以及审阅文件	1. 产品技术要求<br>2. 自检合格报告<br>3. 注册检验合格报告（报告编号：YQZC20180360）<br>4. 临床试验方案（方案编号：FT-YLQX201801；版本号/版本日期 V1.0/2018 年 09 月 26 日）√<br>5. 研究者手册（版本号/版本日期：V1.0/2018 年 08 月 10 日）√<br>6. 知情同意书（版本号/版本日期：V1.1 /20190320）<br>7. 研究者资格证明文件（研究者履历表）<br>8. 病例报告表（版本号/版本日期：V1.0/20180926）<br>9. 临床试验机构的设施条件能够满足试验的综述<br>10. 申办者资质证明文件（企业法人营业执照和医疗器械生产企业许可证）<br>11. 试验用医疗器械的研制符合适用的医疗器械质量管理体系相关要求的声明<br>12. 申办者以及研究者保证所提供资料真实性的声明<br>13. 视觉模拟评分（Visual Analogue Score，VAS）量表<br>14. 产品使用说明书<br>15. 申办者委托函<br>16. 组长单位伦理批件（批件号：南京市中医院伦理委员会 2018NJL020））

<table>
<tr><td>审查意见</td><td colspan="3">根据我国食品药品监督管理局“药物临床试验质量管理规范”(2003 年),“药物临床试验伦理审查工作指导原则”（2010 年），卫计委“涉及人的生物医学研究伦理审查办法”(2016 年)，国家中医药管理局“中医药临床研究伦理审查管理规范”（2010 年），以及世界医学会《赫尔辛基宣言》（2013）等，经本伦理委员会审查，同意按所批准的临床研究方案、知情同意书开展本项研究。</td></tr>
<tr><td>伦理委员会声明</td><td colspan="3">请遵循 GCP 原则、遵循伦理委员会批准的方案开展临床研究，保护受试者的健康与权利。
研究开始前，请申请人尽可能完成临床试验注册。
研究过程中若变更主要研究者，对临床研究方案、知情同意书、招募材料等的任何修改，请申请人提交修正案审查申请。
请按照伦理委员会规定的年度/定期跟踪审查频率，审查人在截止日期前 1 个月提交研究进展报告；申办者应当向组长单位伦理委员会提交各中心研究进展的汇总报告；当出现任何可能显著影响试验进行、或增加受试者危险的情况时，请申请人及时向伦理会员会提交书面报告。
研究纳入了不符合纳入标准或符合排除标准的受试者，符合中止试验规定而未让受试者退出研究，给予错误治疗或剂量，给予方案禁止的合并用药等没有遵从方案开展研究的情况；或可能对受试者的权益/健康以及研究的科学性造成不良影响等违背 GCP 原则的情况，请申办者/监察员/研究者提交违背方案报告。
申请人暂停或提前终止临床研究，请及时提交暂停/终止研究报告。完成临床研究，请提交结题报告。</td></tr>
<tr><td>有效期</td><td>有效期 2 年（自批件下发之日起两年内有效，2019 年 4 月 1 日至 2021 年 4 月 1 日）</td><td>年度/定期跟踪审查频率</td><td>12 个月</td></tr>
<tr><td colspan="2">伦理委员会联系人及联系电话</td><td colspan="2">徐桂琴：0551-62838532</td></tr>
<tr><td colspan="2">伦理委员会主任委员签字</td><td colspan="2">[signature]</td></tr>
<tr><td colspan="2">批准日期</td><td colspan="2">2019 年 4 月 1 日</td></tr>
<tr><td colspan="4">安徽中医药大学第一附属医院（安徽省中医院）医学伦理委员会（盖章）</td></tr>
</table>
